# Phenotypic characterization of a novel Spi1*rs1377416 mouse model of Alzheimer’s Disease

**DOI:** 10.1002/alz.090451

**Published:** 2025-01-03

**Authors:** Giedre Milinkeviciute, Celia Da Cunha, Adrian Mendoza‐Arvilla, Claire Ann Butler, Shimako Kawauchi, Jonathan Neumann, Amber Walker, Andrea J Tenner, Frank M LaFerla, Grant R MacGregor, Kim N. Green

**Affiliations:** ^1^ University of California, Irvine, Irvine, CA USA

## Abstract

**Background:**

Genome‐Wide Association Studies (GWAS) implicate SPI1 (PU.1) as a risk factor for late‐onset Alzheimer’s Disease (LOAD). Within the brain, *SPI1* encodes a microglia‐specific transcription factor, necessary for microglial proliferation and activation. *SPI1* rs1377416 has been identified as a non‐coding GWAS risk variant for AD. We have developed a novel mouse model with the *SPI1* rs1377416 variant to explore its impacts on AD pathogenesis.

**Method:**

By using CRISPR/Cas9, we have generated SPI1*rs1377416 mice that carry a non‐coding mutation corresponding to the rs1377416 SNP found in human *SPI1*. We crossed the SPI1 mice with the 5xFAD mouse model of AD and aged them to 4 and 12 months of age. Coronal brain sections were then obtained and immunolabeled with several markers to visualize amyloid plaques, glial cells and assess axonal and neuritic damage. Confocal images were then obtained and quantified in subiculum and cortex.

**Result:**

We found an age‐related increase in dense core plaque number and size in the subiculum of 5xFAD;SPI1 mice. Microglial volume as well as astrocytic activation were reduced in 5xFAD;SPI1 compared to 5xFAD mice in both brain areas at 12 months. Correspondingly, neuritic dystrophy and axonal damage were also diminished. Significant sex difference was observed in different analyses with males being affected less than females (with the exception of plaque deposition), and mainly detected in subiculum.

**Conclusion:**

Our results indicate that the rs1377416 variant of *SPI1* induces an age‐dependent increase in amyloid deposition in the 5xFAD model of AD. Moreover, this *SPI1* variant exerts a protective effect by suppressing astrocytic response and preventing neuritic and axonal damage. There is a strong sex difference observed between males and females when the variant is present and requires further investigation.

